# Supplementary material for: A new generator for proposing flexible lifetime distributions and its properties
Source: PLoS One. 2020 Apr 29;15(4):e0231908. doi: 10.1371/journal.pone.0231908 (PMC7190131; doi:10.1371/journal.pone.0231908)
Supplement: S1 Appendix — (DOCX) [file pone.0231908.s001.docx]

**Appendix: Probability density functions of the competitors models and data sets.**

We now provide the pdfs of the competitor models. We stick here to the original parameter notations of the introduced models, and hence use the same Greek symbol for different types of parameters in those models.

The pdf of the baseline Fréchet distribution is with scale parameter $\lambda\in\left(0,\infty\right)$and shape parameter $\beta\in\left(0,\infty\right)$.

The pdf of the logarithmic transformed Fréchet distribution is $x\mapsto\frac{\beta\lambda^{\beta}x^{-\beta-1}e^{-\left(\frac{\lambda}{x}\right)^{\beta}}}{\log2\left\{2-e^{-\left(\frac{\lambda}{x}\right)^{\beta}}\right\}}$ with scale parameter $\lambda\in\left(0,\infty\right)$and shape parameter $\beta\in\left(0,\infty\right)$.

The pdf of the Exponentiated Fréchet distribution is with scale parameter $\lambda\in\left(0,\infty\right)$and shape parameters $\alpha,\beta\in\left(0,\infty\right)$.

The pdf of the Marshall-Olkin Fréchet distribution is with scale parameter $\lambda\in\left(0,\infty\right)$ and shape parameters $\alpha,\beta\in\left(0,\infty\right)$.

The pdf of the Kumaraswamy Fréchet distribution is with

scale parameter $\lambda\in\left(0,\infty\right)$ and shape parameters $\alpha,\beta,\theta\in\left(0,\infty\right)$.

**Data 1:** 1.901, 2.132, 2.203, 2.228, 2.257, 2.350, 2.361, 2.396,2.397, 2.445, 2.454, 2.474, 2.518, 2.522, 2.525, 2.532, 2.575, 2.614, 2.616, 2.618, 2.624, 2.659,2.675, 2.738, 2.740, 2.856, 2.917, 2.928, 2.937, 2.937, 2.977, 2.996, 3.030, 3.125,3.139, 3.145,3.220, 3.223, 3.235, 3.243, 3.264, 3.272, 3.294, 3.332, 3.346, 3.377, 3.408, 3.435, 3.493, 3.501,3.537, 3.554, 3.562, 3.628, 3.852, 3.871, 3.886, 3.971, 4.024, 4.027, 4.225,4.395, 5.020

**Data 2:** 0.1, 0.33, 0.44, 0.56, 0.59, 0.72, 0.74, 0.77, 0.92, 0.93, 0.96, 1, 1, 1.02, 1.05, 1.07, 1.07, 1.08, 1.08,1.08, 1.09, 1.12, 1.13, 1.15, 1.16, 1.2, 1.21, 1.22, 1.22, 1.24, 1.3, 1.34, 1.36, 1.39, 1.44, 1.46, 1.53,1.59, 1.6, 1.63, 1.63, 1.68, 1.71, 1.72, 1.76, 1.83, 1.95, 1.96, 1.97, 2.02, 2.13, 2.15, 2.16, 2.22, 2.3,2.31, 2.4, 2.45, 2.51, 2.53, 2.54, 2.54, 2.78, 2.93, 3.27, 3.42, 3.47, 3.61, 4.02, 4.32, 4.58, 5.55

**Data 3:** 0.0301, 0.0384, 0.0630, 0.0849, 0.0877, 0.0959, 0.1397, 0.1616, 0.1699, 0.2137,0.2137, 0.2164, 0.2384, 0.2712, 0.2740, 0.3863, 0.4384, 0.4548, 0.5918, 0.6000,0.6438, 0.6849, 0.7397, 0.8575, 0.9096, 0.9644, 1.0082, 1.2822, 1.3452, 1.4000,1.5260, 1.7205+, 1.9890+, 2.2438+, 2.5068+, 2.6466+, 3.0384, 3.1726+, 3.4411, 4.4219+,4.4356+, 4.5863+, 4.6904+, 4.7808+, 4.9863+, 5.0000+
