# Supplementary material for: A new generator for proposing flexible lifetime distributions and its properties
Source: PLoS One. 2020 Apr 29;15(4):e0231908. doi: 10.1371/journal.pone.0231908 (PMC7190131; doi:10.1371/journal.pone.0231908)
Supplement: S1 Dataset — (DOCX) [file pone.0231908.s002.docx]

**Data 1:**

1.901, 2.132, 2.203, 2.228, 2.257, 2.350, 2.361, 2.396,2.397, 2.445, 2.454, 2.474, 2.518, 2.522, 2.525, 2.532, 2.575, 2.614, 2.616, 2.618, 2.624, 2.659,2.675, 2.738, 2.740, 2.856, 2.917, 2.928, 2.937, 2.937, 2.977, 2.996, 3.030, 3.125,3.139, 3.145,3.220, 3.223, 3.235, 3.243, 3.264, 3.272, 3.294, 3.332,3.346, 3.377, 3.408, 3.435, 3.493, 3.501,3.537, 3.554, 3.562, 3.628, 3.852, 3.871, 3.886, 3.971, 4.024, 4.027, 4.225,4.395, 5.020

**Data 2:**

0.1, 0.33, 0.44, 0.56, 0.59, 0.72, 0.74, 0.77, 0.92, 0.93, 0.96, 1, 1, 1.02, 1.05, 1.07, 1.07, 1.08, 1.08,1.08, 1.09, 1.12, 1.13, 1.15, 1.16, 1.2, 1.21, 1.22, 1.22, 1.24, 1.3, 1.34, 1.36, 1.39, 1.44, 1.46, 1.53,1.59, 1.6, 1.63, 1.63, 1.68, 1.71, 1.72, 1.76, 1.83, 1.95, 1.96, 1.97, 2.02, 2.13, 2.15, 2.16, 2.22, 2.3,2.31, 2.4, 2.45, 2.51, 2.53, 2.54, 2.54, 2.78, 2.93, 3.27, 3.42, 3.47, 3.61, 4.02, 4.32, 4.58, 5.55

**Data 3:**

0.0301, 0.0384, 0.0630, 0.0849, 0.0877, 0.0959, 0.1397, 0.1616, 0.1699, 0.2137,0.2137, 0.2164, 0.2384, 0.2712, 0.2740, 0.3863, 0.4384, 0.4548, 0.5918, 0.6000,0.6438, 0.6849, 0.7397, 0.8575, 0.9096, 0.9644, 1.0082, 1.2822, 1.3452, 1.4000,1.5260, 1.7205+, 1.9890+, 2.2438+, 2.5068+, 2.6466+, 3.0384, 3.1726+, 3.4411, 4.4219+,4.4356+, 4.5863+, 4.6904+, 4.7808+, 4.9863+, 5.0000+
