# Supplementary material for: A new generator for proposing flexible lifetime distributions and its properties
Source: PLoS One. 2020 Apr 29;15(4):e0231908. doi: 10.1371/journal.pone.0231908 (PMC7190131; doi:10.1371/journal.pone.0231908)
Supplement: Attachment — Submitted filename: Response to reviewers.docx [file pone.0231908.s003.docx]

**Answer’s for the comments of reviewers.**

**Reviewers' comments:**

**Reviewer's Responses to Questions**

**Comments to the Author**

1. Is the manuscript technically sound, and do the data support the conclusions? The manuscript must describe a technically sound piece of scientific research with data that supports the conclusions. Experiments must have been conducted rigorously, with appropriate controls, replication, and sample sizes. The conclusions must be drawn appropriately based on the data presented.

Reviewer #1:Yes

Reviewer #2: Partly

2. Has the statistical analysis been performed appropriately and rigorously?

Reviewer #1: Yes

Reviewer #2: Yes.

Have the authors made all data underlying the findings in their manuscript fully available?

The [PLOS Data policy](http://www.plosone.org/static/policies.action#sharing) requires authors to make all data underlying the findings described in their manuscript fully available without restriction, with rare exception (please refer to the Data Availability Statement in the manuscript PDF file). The data should be provided as part of the manuscript or its supporting information, or deposited to a public repository. For example, in addition to summary statistics, the data points behind means, medians and variance measures should be available. If there are restrictions on publicly sharing data—e.g. participant privacy or use of data from a third party—those must be specified.

Reviewer #1: Yes

Reviewer #2: Yes

3. Is the manuscript presented in an intelligible fashion and written in standard English?
   PLOS ONE does not copyedit accepted manuscripts, so the language in submitted articles must be clear, correct, and unambiguous. Any typographical or grammatical errors should be corrected at revision, so please note any specific errors here.

Reviewer #1: Yes

Reviewer #2: Yes

**Reviewer #1**: This paper proposes a new generator for flexible lifetime distributions and discusses its statistical properties. Its performance is demonstrated by a Monte Carlo simulation study and three real-life applications. The paper is generally well structured and easy to access. A limitation is that the comparison between the proposed model and other alternatives only focuses on the goodness-of-fit and the results of AIC suggest that the difference seems to be insignificant. From the perspective of practical application, the model complexity should also be considered. The authors are suggested to conduct the model comparison using Bayesian methods. The deviance information criterion available in Bayesian inference, which is deemed as a generalization of the AIC, provides a combined measure of model fit and complexity. Please refer to some representative works which also model the censored continuous variables as in this paper, including:

A Bayesian spatial random parameters Tobit model for analyzing crash rates on roadway segments. Accident Analysis and Prevention, 2017, 100: 37-43.

A multivariate random parameters Tobit model for analyzing highway crash rate by injury severity. Accident Analysis and Prevention, 2017, 99: 184-191.

Incorporating temporal correlation into a multivariate random parameters Tobit model for modeling crash rate by injury severity. Transportmetrica A: Transport Science, 2018, 14 (3): 177-191.

Jointly modeling area-level crash rates by severity: A Bayesian multivariate random-parameters spatio-temporal Tobit regression. Transportmetrica A: Transport Science, 2019, 15(2): 1867-1884.

**Answer**: As per suggestion of the learned reviewer, we have calculated DIC and reported the results along with the results of AIC and other goodness of fit statistics for all three considered data sets. Moreover, Bayes estimates and posterior standard deviations are also reported. Also, some relevant papers reporting DIC as a goodness of fit criterion have been cited at appropriate places.

**Reviewer #2**: The topic of this paper is interesting and the methods sound. There are several suggestions to improve this paper.

1. “we will take the Fréchet as example” could be “we will take the Fréchet distribution as example”. And some references are needed.

**Answer**: corrected as suggested. Relevant reference articles have also been mentioned.

2. “Typically this requires numerical optimization techniques such as Newton-Raphson.” References are needed for these techniques.

**Answer**: References have been included.

3. This paper lacks of references in the last decade.

**Answer**: Yes! We agree with the referee. Unfortunately, we could not find any major transformer or technique related to this topic. Most of the recent work is based on extensions of previously proposed methods. We have included only major/ well known methods available in the literature. It is to be noted that only those frechet distributions which are generated from well known transformations are considered for comparison purposes using different data sets. As far as censoring schemes are concerned, we have included more recent work.

4. “The score equations, and associated maximum likelihood estimates, are obtained along the same lines as in the previous sections, hence we leave this to the reader.” This sentence is not suitable. Some reference could be added instead of letting reader guess. For example, the following ones.

[1] Investigation on the Injury Severity of Drivers in Rear-End Collisions Between Cars Using a Random Parameters Bivariate Ordered Probit Model, International Journal of Environmental Research and Public Health, 2019, 16(14) , 2632.

[2] Injury severities of truck drivers in single- and multi-vehicle accidents on rural highway, Accident Analysis and Prevention, 2011, 43(5), 1677-1688.

[3] Analysis of hourly crash likelihood using unbalanced panel data mixed logit model and real-time driving environmental big data. 2018, JOURNAL OF SAFETY RESEARCH. 65: 153-159.

[4] Investigating the Differences of Single- and Multi-vehicle Accident Probability Using Mixed Logit Model, Journal of Advanced Transportation, 2018, UNSP 2702360.

**Answer**: Actually, this line was written for the sake of brevity. Obviously, score equations can be obtained by taking first derivative of log-likelihood function with respect to the parameters and then equating to zero. In earlier draft of the paper, we did not include these simple equations to save the space. To support the brevity, related reference papers have been included now in the revised manuscript.

5. For AIC, these references could also be referred to.

**Answer**: The mentioned reference papers have been included.

6. There are some ◇ in this paper, which might be not correct. For example, Page 10.

**Answer**: corrected as mentioned.

7. For censored data, Tobit models are frequently used, which could be referred to the following papers.

[5] Modeling crash rates for a mountainous highway using refined-scale panel data”, Transportation Research Record, 2015, 2515:10-16.

[6] Refined-scale panel data crash rate analysis using random-effects tobit model, Accident Analysis and Prevention, 2014, 73, 323-332.

**Answer**: Relevant papers discussing tobit models have been cited.

8. “a p-value of 0.02663451” could be “a p-value of 0.027”.

**Answer**: Changed by rounding all the results on p-values to four decimal places.
